# Prognostic value of CD44 expression in patients with hepatocellular carcinoma: meta-analysis

**DOI:** 10.1186/s12935-016-0325-2

**Published:** 2016-06-17

**Authors:** Yangkun Luo, Yan Tan

**Affiliations:** Department of Radiation Oncology, Sichuan Cancer Hospital, No.55, Section 4, Renmin South Road, Chengdu, 610041 Sichuan China

**Keywords:** CD44, Prognosis, Meta-analysis, Overall survival, Disease-free survival

## Abstract

**Background:**

CD44 has been reported to be involved with tumor growth and metastasis and has also been implicated as a CSC marker in hepatocellular carcinoma (HCC). However, the prognostic value of CD44 still remains controversial; hence, we investigated the correlation between CD44 and the clinicopathological features of HCC by meta-analysis.

**Methods:**

Identification and review of publications assessing clinical or prognostic significance of CD44 expression in HCC until November 1, 2015. A meta-analysis was performed to clarify the association between CD44 expression and clinical outcomes.

**Results:**

A total of 14 publications met the criteria and comprised 2235 cases. Analysis of these data showed that CD44 expression was not significantly associated with the tumor differentiation (OR 1.48, 95 % confidence interval [CI] 0.85–2.60, P = 0.17), AFP level of HCC patients (OR 0.83, 95 % CI 0.52–1.33, P = 0.45), or disease-free survival (relative risk [RR] 1.15, 95 % CI, 0.85–1.54; P = 0.36). However, in the identified studies, CD44 expression was highly correlated with tumor TNM classification (OR 2.38, 95 % CI 1.23–4.60; P = 0.01) and decreased overall survival (RR 1.49, 95 % CI, 1.26–1.76; P < 0.00001).

**Conclusions:**

This meta-analysis shows CD44 expression in HCC is connected with decreased overall and thus marks a worse prognosis.

## Background

Hepatocellular carcinoma (HCC) is the fifth most common cancer worldwide and the most common malignant primary tumor in the liver [1]. Despite improvements in treatment modalities during the past few decades, the prognosis of HCC is still very poor because of frequent intrahepatic metastasis and tumor recurrence [2]. It is, therefore, desirable to develop a deeper understanding of the biology of this disease to adapt current therapeutic strategies and to develop therapies that are more effective. Evidence has recently been accumulating to support the hypothesis that solid tumors contain a small subpopulation of cells called cancer stem-like cells (CSC), which exhibit self-renewing capacities and are responsible for tumor maintenance and metastasis [3] and possibly for resistance towards chemotherapy and radiation therapy [4]. Thus, it is of major importance to investigate CSCs associated with cancer progression as they may be important factors in determining the clinical outcomes of cancer. Furthermore, CSC markers, such as CD44, CD133 and ALDH1, are potential indicators of HCC prognosis [5–7]. Among these CSC markers, CD44 is the most frequently reported in HCC. Several studies similarly utilised CD44 positivity to isolate cells with stem cell-like and cancer-initiating properties from other cancer cells [8–10].

The CD44 receptor is a typeItransmembrane glycoprotein that was initially identified as a leukocyte antigen [11]. The alternative splicing of variable exons of CD44 results in abundant variants, which are denoted CD44v, and the isoform with no variable exons in the mRNA is named CD44 standard (CD44s) [12]. The smallest, standard isoform is CD44s, which is generally expressed on vertebrate cells, while CD44v is only expressed on some epithelial cells [13]. CD44 is the major hyaluronan (HA) receptor [13], and CD44 bound to HA has been proven to participate in various tumor biological activities, including tumor progression, metastasis and proliferation [14, 15].

However, the correlations between CD44 and clinicopothological features of HCC and theirs prognostic values are still controversial. Some concluded that CD44 expression had no influence on survival [16, 17]. While others reported that CD44 expression was predictive of decreased survival outcome for HCC [18–20]. In order to evaluate this question, we conducted a systematic review and meta-analysis to determine the association between the expression of HCC and clinicopathological features and prognosis of HCC patients.

## Methods

### Search strategy

A systematic literature search was performed in the electronic databases Pubmed, Embase and Wanfang until November of 2015. Search terms included “CD44”, “hepatocellular carcinoma” or “HCC” or “liver cancer” or “liver tumor” or “liver neoplasms” or “hepatocellular carcinoma”. The titles and abstracts of potential references were carefully scanned to exclude irrelevant articles. The remaining articles were evaluated to identify research that contained the topic of interest, and full texts were reviewed in depth afterwards.

### Selection criteria

The studies included in the present meta-analysis were randomised controlled studies or observational studies (case–control or cohort) that evaluated the association between the expression of CD44 and the prognosis or the clinical data of HCC. Studies were included if they (1) focused on the clinical features or prognosis of HCC; (2) analysed the correlation of CD44 expression with clinical features and survival outcomes [(disease-free survival (DFS) or overall survival (OS)]. Articles were excluded on the basis of the following criteria: (1) non-association studies; (2) review articles or case reports; (3) association studies for other diseases; (4) overlapped research; (5) insufficient data on survival.

All evaluations were independently performed by two reviewers to ensure the accurate inclusion of studies. When several studies containing overlapping data emerged, the one with the largest data set was adopted. If the original data were not provided in the text, we contacted the authors for the data necessary to conduct the meta-analysis.

### Data extraction

All data were extracted by two independent reviewers. Disagreements in data extraction were resolved by reaching a consensus in accordance with the original article. The following relevant data were extracted in a predefined table: author, publication year, patient’s country, tumor stage, number of patients, research technique used and the choice of cutoff scores for the definition of positive staining or staining intensity. Considering that some studies displayed survival data with a Kaplan–Meier curve, we used GetData Graph Digitizer 2.26 (http://getdata-graph-digitizer.com/) to digitise and extract survival data.

### Statistical analysis

Stata version 12.0 (StataCorp LP, TX) was used in this meta-analysis. The statistical analysis was performed according to the guidelines proposed by the meta-analysis of observational studies in Epidemiology group. ORs with 95 % CI were used to evaluate the correlation between CD44 expression and clinicopathological features, including tumor TMN classification, tumor grade and AFP level in HCC patients. RRs with 95 % CI were used to evaluate correlation between CD44 expression and 5-year DFS and 5-year OS. The heterogeneity among studies was measured using the Q test and I2 test. A fixed-effects model was used in the absence of significant heterogeneity; a random-effect model was used otherwise. Begg’s and Egger’s tests were performed to identify the potential publication bias. P <  0.05 was considered to indicate statistical significance. All P values are two-tailed. Sub-group analyses were performed to investigate the association of CD44 expression with clinical features and prognosis in terms of method, cutoff of staining, sample size, follow-up time and subtypes of the CD44 family. Furthermore, a sensitivity analysis was performed to examine the robustness of the pooled results.

## Results

### Description of studies

**Fig. 1:**
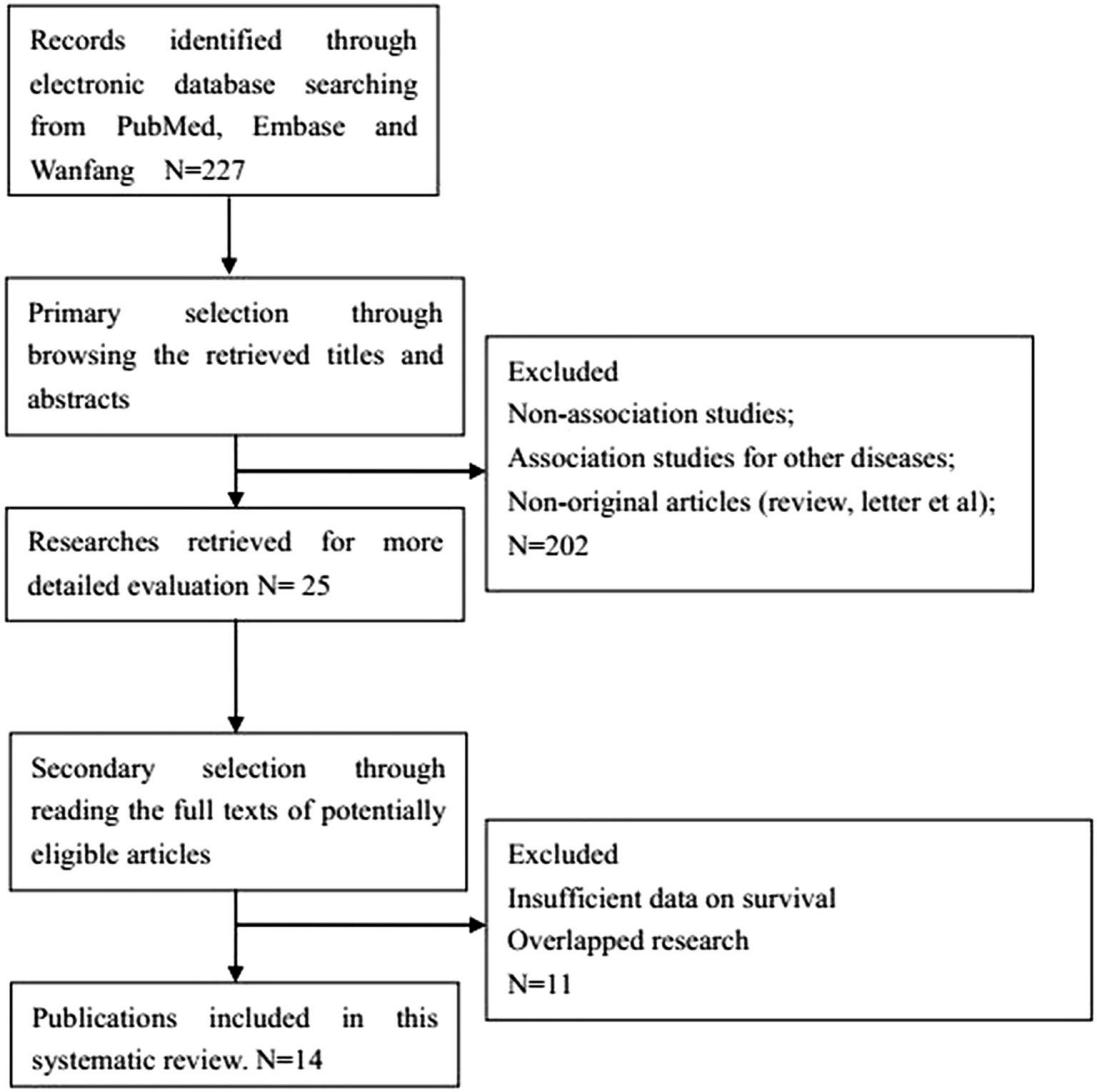
Literature search strategy and selection of articles

A total of 227 articles were selected for the meta-analysis by browsing the databases PubMed, Embase and Wanfang, of which 202 were excluded after reviewing the title and abstract, eleven articles were excluded after reviewing the full publications (Fig. 1). Eventually, a total of 14 studies with 2235 patients, who fulfilled all of the inclusion criteria, were considered for the analysis [16–29]. All patients in the eligible studies were determined by pathological stage. All of the studies reported the prognostic value of CD44 expression for survival in patients with HCC. Estimation using survival curves were segregated according to either DFS or OS. A RR on DFS or OS could be extracted for all enrolled studies. The characteristics of the included studies are listed in Table 1.

**Table 1:** Characteristics of the included studies

Study	Patient’s country	Ethnicity	Year	Time of collection	Pathological stage	Method	Number of patients	Age in years	Follow-up months	Cut-off for CD44 positive	Survival analysis
Endo et al. [18]	Japan	Asian	2000	ND	ND	IHC	107	17–80	80	>0 % staining	OS
Huang et al. [22]	China	Asian	2001	1995–1999	I–IV	IHC	51	36–72	48	>0 % staining	OS
Su et al. [27]	China	Asian	2006	1996–1999	I–IV	IHC	40	32–70	36	>0 % staining	OS
Yang et al. [28]	China	Asian	2008	1997–2000	I–IV	IHC	302	26–75	121	>10 % staining	OS; DFS
Zhang et al. [21]	China	Asian	2008	2001–2004	I–IV	IHC	50	21–68	ND	>20 % staining	OS
Peng et al. [23]	China	Asian	2010	2005–2006	ND	IHC	76	19–69	36	>25 % staining	OS
Ryu et al. [17]	Korea	Asian	2011	1990–2003	I–IV	IHC	260	ND	137	>10 % staining	OS; DFS
Tovuu et al. [25]	Japan	Asian	2012	2005–2009	I–IV	RT–PCR	48	ND	48	Median	OS; DFS
Mima et al. [16, 19]	Japan	Asian	2012	2004–2007	I–IV	IHC	150	ND	60	>0 % staining	OS; DFS
Zhou et al. [24]	China	Asian	2012	2007–2010	I–III	RT–PCR	323	ND	74	Median	OS; DFS
Mima et al. [16, 19]	Japan	Asian	2012	2004–2007	I–IV	IHC	235	ND	60	>50 % staining	OS; DFS
Chen et al. [26]	China	Asian	2014	2005–2008	I–IV	IHC	387	ND	96	>10 % staining	OS
Cao et al. [29]	China	Asian	2014	2005–2011	ND	IHC	46	22–79	85	>25 % staining	OS
Hu et al. [20]	China	Asian	2014	2006–2007	I–III	IHC	160	29–72	84	>10 % staining	OS; DFS

### Correlation of CD44 expression with clinicopathological parameters

**Fig. 2:**
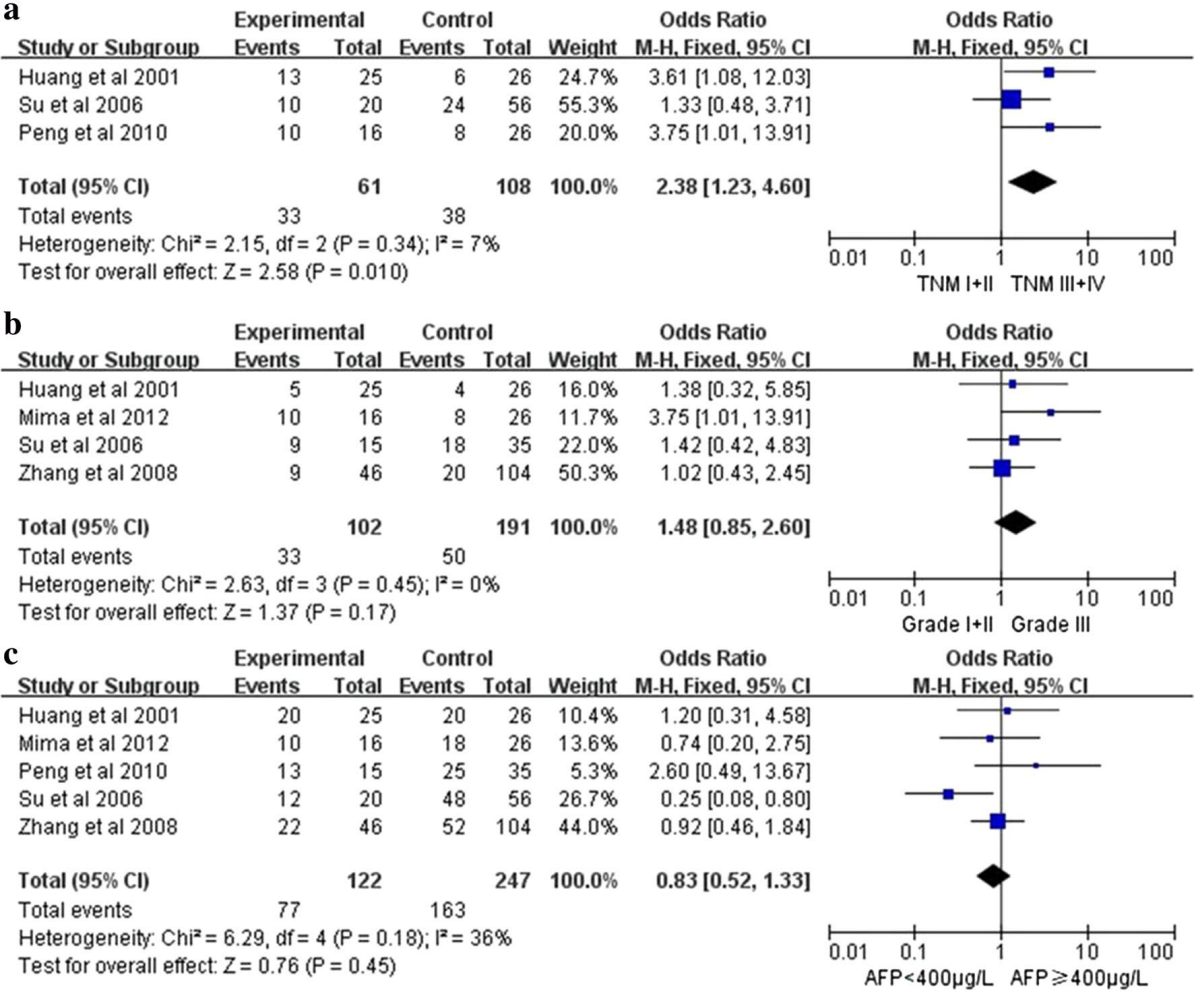
Forest plot depiction of CD44 expression and OR for clinical pathologic features. Clinicopathological parameters investigated are (**a**) TMN classification, (**b**) tumor grade (**c**)AFP level

The association between CD44 and several clinicopathological parameters is illustrated in Fig. 2. CD44 expression was highly correlated with tumor TNM classification (I+II vs. III+IV)(OR 2.38, 95 % CI 1.23–4.60, P = 0.01, fixed-effect) (Fig. 2a). However, CD44 expression was not significantly associated with tumor differentiation (G1 + G2 vs. G3) (OR 1.48, 95 % CI 0.85–2.60, P = 0.17, fixed-effect) (Fig. b) and AFP level of HCC patients (OR 0.83, 95 % CI 0.52–1.33, P = 0.45, fixed-effect) (Fig. 2c).

### CD44 expression and 5-year survival outcome

**Fig. 3:**
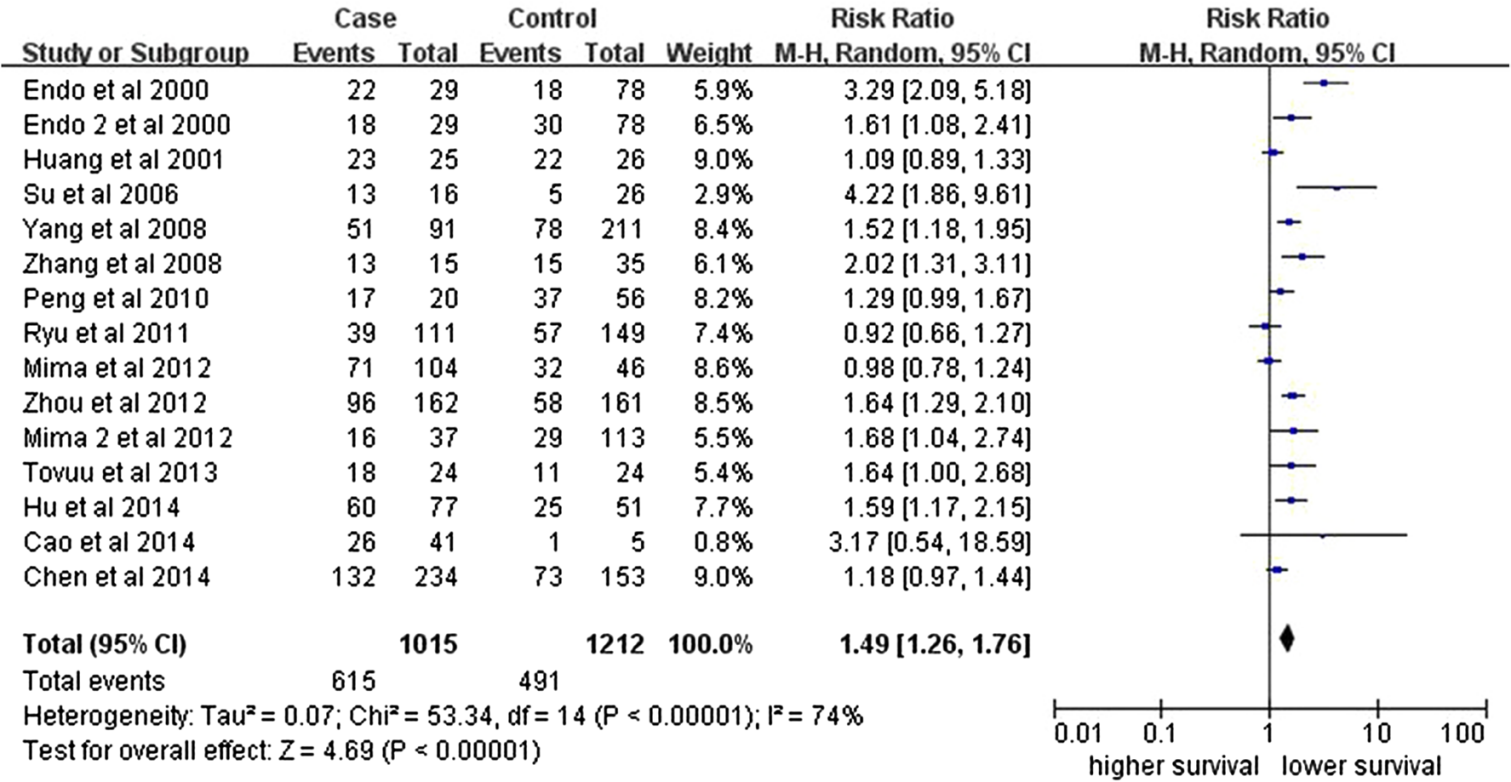
Forest plot illustrates the association between CD44 expression and OS of HCC

Fourteen studies investigating OS were pooled into the meta-analysis. As shown in Fig. 3, there was significant association between CD44 expression and OS (RR, 1.49, 95 % CI, 1.26–1.76; P < 0.00001) with significant heterogeneity (I2 = 74 %, P < 0.0001). We also performed subgroup analysis by method, cutoff of staining, sample size, follow-up time or subtypes of the CD44 family in HCC. Significant association was also detected in all stratified analysis (Table 2).

**Table 2:** Associations between CD44 expression and OS of HCC grouped by selected factors

Stratified analysis	Studies	Odds ratio		Model	Heterogeneity	
		OR (95 % CI)	POR		I2 (%)	P
OS	15	1.49 (1.26–1.76)	<0.00001	Random	74	<0.00001
Method						
IHC	13	1.48 (1.22–1.78)	<0.0001	Random	76	<0.00001
RT-PCR	2	1.64 (1.32–2.04)	<0.0001	Fixed	0	0.99
Cut off						
>10 %	3	1.63 (1.05–2.54)	0.03	Random	55	0.11
≤10 %	10	1.45 (1.17–1.80)	0.0007	Random	80	<0.00001
Median	2	1.64 (1.32–2.04)	<0.0001	Fixed	0	0.99
Sample size						
>50	11	1.38 (1.17–1.64)	0.0002	Random	75	<0.0001
≤50	4	2.26 (1.64–3.12)	<0.00001	Fixed	30	0.23
Follow-up(m)						
>60	8	1.53 (1.22–1.92)	0.0002	Random	74	0.0004
≤60	7	1.46 (1.12–1.90)	0.005	Random	76	0.0004
Subtypes of the CD44 family						
CD44	4	1.35 (1.16–1.56)	0.0001	Fixed	24	0.27
CD44v6	6	1.51 (1.11–2.05)	0.008	Random	86	<0.00001
CD44s	5	1.59 (1.10–2.29)	0.01	Random	74	0.004

*POR* P value for odds ratio

### CD44 expression and DFS in HCC

**Fig. 4:**
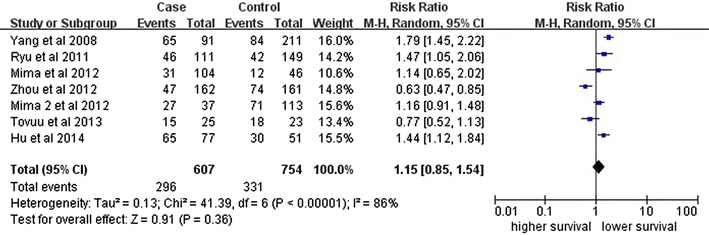
Analysis of CD44 expression and DFS among included studies

Seven studies identifying DFS were pooled into the meta-analysis. As shown in Fig. 4, there was no significant association between CD44 expression and DFS (RR, 1.15, 95 % CI, 0.85–1.54; P = 0.36) with significant heterogeneity (I2 = 86 %, P < 0.00001).

In fact, three out of seven studies have concluded CD44 expression as an unfavorable prognostic factor in HCC patients.

### Publication bias

**Fig. 5:**
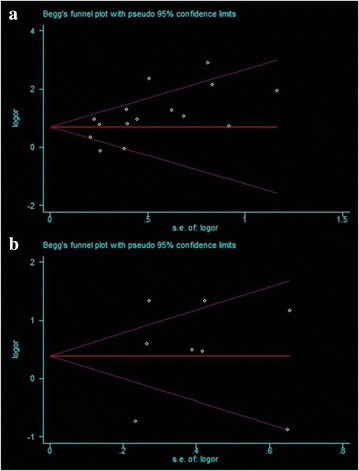
Begg’s funnel plot estimated the publication bias of the included literature for OS (**a**) and DFS (**b**)

The funnel plots presented no evidence of publication bias in the studies of either outcome (Fig. 5a, b). No evidence for significant publication bias was found in OS (Egger’s test, P = 0.151) and DFS (Egger’s test, P = 0.805) studies.

### Sensitivity analysis

**Fig. 6:**
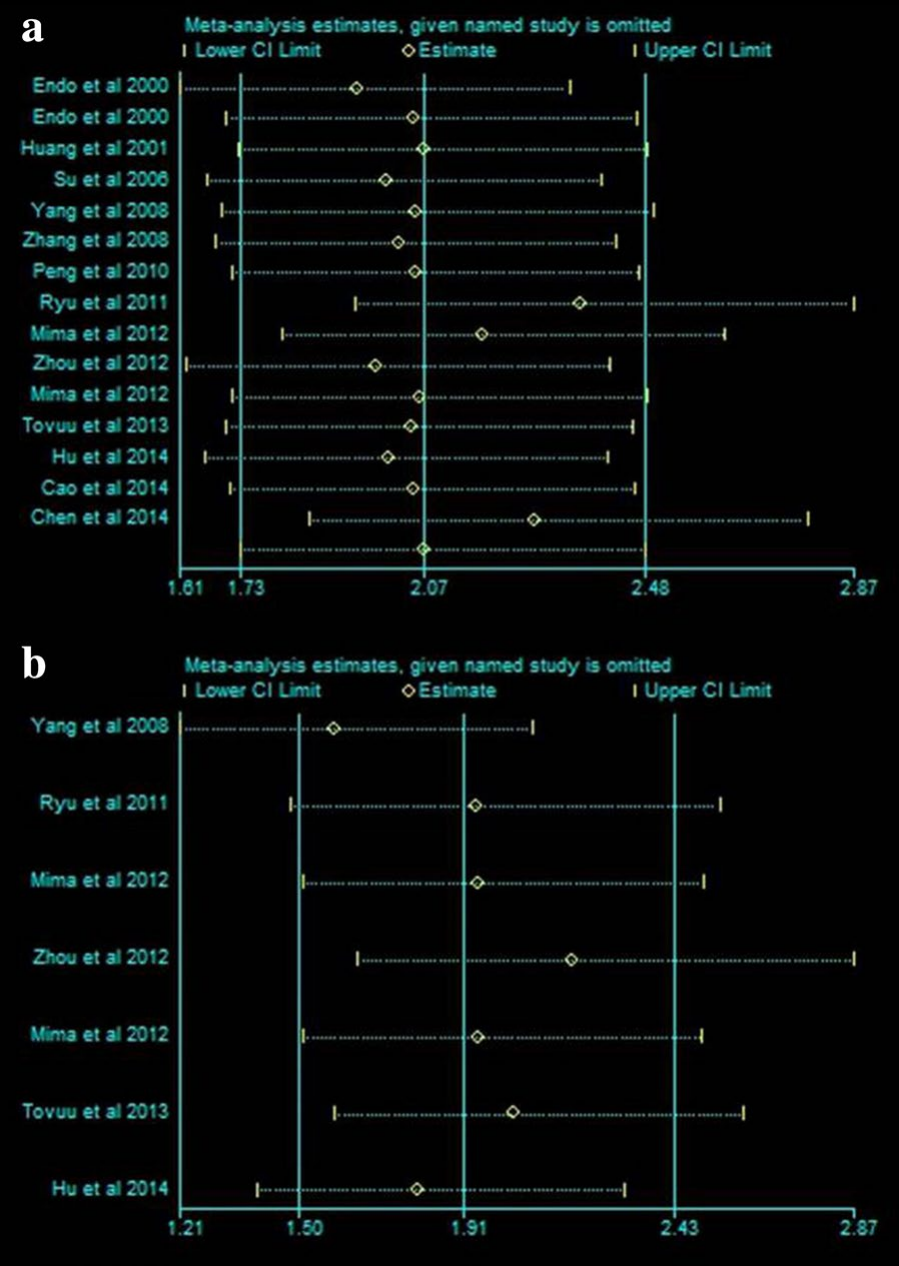
Sensitivity analysis of all the studies assessing OS (**a**) and DFS (**b**)

In order to test for a bias introduced by the low numbers of available eligible publications we performed a sensitivity analysis. For this a single study involved in the meta-analysis was omitted for each round of analysis to investigate the influence of the individual data set of the particular study to the pooled ORs. We found that that pooled RRs of OS and DFS was not significantly changed, suggesting the robustness of our results (Fig. 6a, b).

## Discussion

To the best of our knowledge, this is the first meta-analysis of published studies to evaluate the association between total CD44 expression (including CD44, CD44 s and CD44v6) and prognosis in HCC. Heterogeneity analysis and sensitivity analysis were also critically performed to ensure the epidemiological credibility of this meta-analysis. The present results indicate that CD44 expression is positively associated with higher tumor TNM stage, as well as poor OS for patients with HCC. However, we find no significant association between CD44 positivity and differentiation degree, AFP level or DFS for patients with HCC. This trend suggests that CD44 can function as a prognostic factor for predicting the outcomes of HCC patients. Therefore, our data imply that elevated CD44 expression can contribute to HCC development and progression, and the detection of the CD44 aberrations may be useful for identifying poor prognoses in patients with HCC. For future studies, co-expression of hepatic CSC markers associated with patient survival may be more meaningful for clinical application in HCC. Several studies have shown that CSC-related factors, including CD133, EPCAM, and CD90, are associated with HCC progression [30–32]. In addition, CSCs have major phenotypic and functional heterogeneity which may help distinguish them from cancer cells, and may be of potential benefit in the development of anti-cancer therapies to improve clinical outcomes.

A previous study showed that CD44-positive tumor cells have CSC properties, such as self-renewal and tumorigenicity [33]. Expression of CD44 has been closely linked to tumor progression, metastasis, and treatment resistance processes in various cancers [34–36]. In particular, CD44 is closely associated with aggressive behavior and correlates with poor prognosis in a variety of human malignancies, and it has been shown to regulate malignant transformation by inducing tumor cell proliferation, adhesion, and migration [37]. Furthermore, the prognostic value of CD44 expression in HCC is yet to be elucidated. The results of our study indicated that increased CD44 expression correlated with poor OS of HCC patients. However, contrasting results were also reported. For instance, Horiguchi et al. reported that CD44 expression correlates with favorable prognosis in breast cancer [38]. In addition, Liang et al. reported that gastrointestinal stromal tumor patients with higher expressions of CD44 survived longer and had lower recurrence rates [39]. These conflicting results suggest an elusive role of CD44 in cancer progression and metastasis. Thus, more prospective studies are needed to draw a definite conclusion.

Although our study revealed the positive correlation between CSCs marker CD44 and tumor TNM stage and survival of patients with HCC, CD44 itself as a biomarker has its limitations on predicting prognosis and clinicopathological parameters in patients. Firstly, overall survival (OS) and disease free survival (DFS) were determined from unadjusted RRs in the published papers, and RRs from the survival curves might be less reliable those that from direct analysis of variance. Ideally, measurements should be directly obtained from the statistical data in published papers and then adjusted by using other prognostic factors. Secondly, in the assessment of biomarkers, the use of a standard threshold is of great importance. Although immunohistochemistry was the most commonly applied method, differences in cut-off values for positive CD44 expression may have contributed to the observed heterogeneity. Thirdly, the OR of each study is generally small and the conclusion might be affected by one or two reports with large ORs. All of these factors might partly influence the significance of CD44 expression in the survival and the clinicopathological analysis. Fourth, the survival data are achieved directly, calculated from the available data, or extracted from the K-M curves in the articles. The latter two methods are less reliable than direct analysis of primary data [40].

In summary, this meta-analysis indicated that CD44 expression was associated with tumor TNM stage in HCC. Moreover, positive CD44 expression was associated with a worse outcome than CD44-negative expression, and CD44 was an independent factor associated with reduced survival. The relative simplicity in the methodology for using CD44 expression for the identification of hepatic CSC suggests that this marker should be further evaluated for their potential use in the identification of hepatic CSC in clinical practice.
